# Perspectives on Participation in Continuous Vocational Education Training–An Interview Study

**DOI:** 10.3389/fpsyg.2020.01096

**Published:** 2020-06-30

**Authors:** Christin Siegfried, Josephine Berger

**Affiliations:** ^1^Faculty of Economics and Business, Business Education, Goethe-University Frankfurt, Frankfurt, Germany; ^2^Institute for General Education and Vocational Education, Technical University of Darmstadt, Darmstadt, Germany

**Keywords:** continuous vocational education training, disadvantaged groups, reasons and barriers, interview study, employers, employees, jobseekers

## Abstract

In European industrialized countries, a large number of companies in the healthcare, hotel, and catering sectors, as well as in the technology sector, are affected by demographic, political, and technological developments resulting in a greater need of skilled workers with a simultaneous shortage of skilled workers (CEDEFOP, 2015, 2016). Consequently, employers have to address workers who have not been taken into account such as low-skilled workers, workers returning from a career break, people with a migrant background, older people, and jobseekers and train them, in order to guarantee the professionalization of this workforce (Festing and Harsch, 2018). Continuing vocational education and training (CVET) is seen as an indispensable tool; because CVET has advantages for both employers and employees, it helps to increase the productivity of companies (Barrett and O’Connell, 2001), to prevent the widening of socioeconomic disparities (Dieckhoff, 2007), and to open up career opportunities for the workforce (Rubenson and Desjardins, 2009). However, participation rate on CVET seems to differ, depending on institutional factors (such as sector and size of the company) and individual characteristics (such as qualification level, migration background, age and time of absence from work) (e.g., Rubenson and Desjardins, 2009; Wiseman and Parry, 2017). In contrast to previous research, our study aims to provide a holistic view of reasons for and against CVET, combining the different perspectives of employers and (potential) employees. The analysis of reasons and barriers was carried out based on semi-structured interviews. Fifty-seven employers, 73 employees, and 42 jobseekers (potential employees) from the sectors retail, healthcare and social services, hotels and catering, and technology were interviewed. Results point to considerable differences in the reasons and barriers mentioned by the disadvantaged groups. These differences are particularly significant between employees on the one side and employers, as well as jobseekers, on the other side, while the reasons to attend CVET of jobseekers are more similar to those of employers. The results can be used to tailor CVET more closely to the needs of (potential) employees and thus strengthen both the qualification and career opportunities of (potential) employees and the competitiveness and productivity of companies.

## Introduction

As the rationalization of human work is becoming a focus, a shift from manufacturing companies to service providers is expected and will make simple help activities less necessary (Bellmann et al., 2014; Mönnig et al., 2019). This structural change is being accelerated by digitization and has fundamental consequences for the human resource planning of companies (e.g., Gebhardt et al., 2015; Frey and Osborne, 2017; Fischer et al., 2018; Harteis, 2018; Harteis et al., 2020). Thus, more information technology positions have to be filled, such as in the software and application development or artificial intelligence, for which there is already a great demand on the labor market. Moreover, for employees in almost all industries and types of work, digitization modifies job requirements of their tasks, so that they need new skills and abilities. Furthermore, digitization can be used to optimize routines and processes and to partially or completely automate work performance. Otherwise, work tasks will become more focused on monitoring and managing such digitalized work processes, which in turn requires new corporate strategies and changes in the organizational structure, such as flatter hierarchies.

This organizational and technological changes require a continuous adaptation of the human resources, and the skills and abilities of employees must adapt accordingly (e.g., Bellmann et al., 2014; Gebhardt et al., 2015; Harteis, 2018; Harteis et al., 2020). On the other hand, companies face the risk of shortages of skilled labor due to demographic developments (CEDEFOP, 2015; Paul, 2016). Thus, the competition among employers for an adequately qualified workforce will intensify. In particular, the retail, healthcare, hotel and catering, and technology sectors will be or are already affected (CEDEFOP, 2016). Opportunities to overcome the shortage of skilled workers include the usage and integration of a previously unused potential workforce by increasing labor migration from abroad and qualifying that workforce, and/or even qualifying one’s own disadvantaged workforce, through appropriate continuing vocational education and training (CVET) (Paul, 2016; Festing and Harsch, 2018). Furthermore, CVET is seen to have a positive effect on the productivity and profitability of a company, whereby its market value and competitiveness can be improved (Zwick, 2005; Kuckulenz, 2007). At the same time, CVET for employees, and also for potential employees in the form of jobseekers, offers an important opportunity for their professional (e.g., increase in individual wages) and individual development (e.g., reduce the risk of becoming jobseekers) (Barrett and O’Connell, 2001; Dieckhoff, 2007; Rubenson and Desjardins, 2009; Friebe and Schmidt-Hertha, 2013).

For this reason, CVET is seen as a collective good, from which all participants in the labor market, as well as the state and society, benefit equally (Becker and Hecken, 2009; Rubenson and Desjardins, 2009; CEDEFOP, 2015). Although this relationship between CVET participation and the achievement of the desired effect is greatly simplified and does not take into account a number of factors influencing the learning transfer, such as the quality or suitability of the CVET, the working environment in which the new knowledge has to be applied, or trainee characteristics (see Hinrichs, 2014; Sandmeier et al., 2018; Harteis et al., 2020), access to CVET is an important basis for any learning process. Therefore, research results that point to considerable disparity in CVET participation are critical. It appears that CVET participation and its rate of being offered are largely heterogeneous regarding different personal and firm characteristics, and this demonstrates that especially older people, people with a migrant background, people re-entering the workforce, low-formally qualified people, and persons employed at smaller companies, in retail, healthcare, hotel and catering, and technology sectors are prone to lower participation rates in CVET (Chrisholm et al., 2004; Bassanini et al., 2007; Rubenson and Desjardins, 2009; Bellmann et al., 2010; Desjardins, 2014).

This issue gives rise to various questions focusing especially on the institutional context regarding decision-making and steering processes, the examination of influencing factors of CVET participation, and also on a review of the interests of the actors involved (e.g., Käpplinger, 2016).

In the context of this study, the actor-related points of view in particular are of special interest by comparing the perspective and thus the interests of the (potential) employee and the employer. The research interest refers to findings regarding possible discrepancies and also to commonalities of CVET actors regarding the reasons and barriers to offering or participating in CVET.

For this purpose, see Section “Theoretical Framework” with theoretical explanatory models of participation in CVET and offering of CVET on the part of employers and presents empirical findings on reasons and barriers to do so by employees, jobseekers, and employees. See Section “Empirical Findings on Reasons for and Barriers to Attending or Offering CVET by (potential) Employees and Employers” presents the study and its results. The paper concludes with a discussion of the results and an outlook.

## Theoretical Framework

### Continuing Vocational Education and Training

In the context of CVET, six essential functions can be referred to Bohlinger and Münk (2008, pp. 66f.): first, an adoption function to be able to react to a changing economic, as well as social and technological changes by promoting individual development; second, an innovation function whereby CVET should contribute to increasing productivity by upgrading the knowledge of employees; third, a promotion function to form careers; fourth, a catch-up function in the absence of initial professional training; fifth, a compensation function in order to offer new career options; and sixth, a preventive function to keep a current occupation or current position in a job.

To achieve these goals, there are different CVET formats and types that can be distinguished. Thus, a CVET can be formal, non-formal, and informal (Eshach, 2007; Bilger and Kuper, 2017, p. 17). Formal education covers the area of regular educational activities, such as school, training, and also master courses (Eshach, 2007). Another characteristic of formal continuing education is the acquisition of a recognized qualification that is linked to a National Qualifications Framework. Informal learning can be equated with the concept of self-learning and is neither managed nor certified by an institution (Eshach, 2007). Non-formal education refers to courses that are not provided by a regular educational institution (Eshach, 2007). Non-formal education includes, for example, software training and language courses; the courses contain a recognizable curriculum, but they are not officially certified either.

Regarding the differentiation of CVET with respect to its type (Authoring Group Educational Reporting, 2016; Bilger and Kuper, 2017, p. 26; see also Seeber et al., 2017), general, vocational, and political education can be named. However, because all different types and formats of CVET can support the achievement of the various functions of CVET, it seems to be helpful to initially include all formats and types in the first instance in this study.

### Differential Explanatory Approaches to Offering and Participating in CVET

While there are many different approaches to explain disparities in participation in CVET (see Stocké et al., 2011; Desjardins, 2014), the focus of empirical studies is on the theory of human capital (e.g., Zwick, 2005; Neubäumer and Kohaut, 2007; Walden, 2007; Sala and Silva, 2013; Kilpi-Jakonen et al., 2015; Osiander and Dietz, 2016). Occasionally, the framework of discussion is expanded to include arguments referring to the rational choice theory, transaction cost theory or signaling theory (Zwick, 2005; Kilpi-Jakonen et al., 2015; Wotschack, 2019). This also applies to explanatory approaches that focus more on participation in CVET as an individual educational decision, for example, of the (potential) employee such as the expectation-value theory (e.g., Van den Broeck et al., 2010; Gorges and Kandler, 2012; Gorges, 2015, 2016). In order to use these diverse approaches to discuss reasons and barriers to participate in CVET and incorporate the findings of the aforementioned studies, these explanatory approaches are briefly explained in the following.

According to the investment hypothesis underlying the human capital theory, educational activities are investments that generate both costs and returns (Becker, 1994). In this connection, CVET should be regarded as a human capital investment decision (Becker, 1994). From an employer’s perspective, CVET of an employee is only worthwhile if the costs incurred are offset by increased labor productivity in the future, which in turn exceeds the costs (Nafukho et al., 2004). While the human capital theory disposes of complete information, the filter or signaling theory, according to Arrow (1973) or Spence (1974), places uncertainty in the foreground. From an employer’s point of view, the aim is to avoid possible misplacements, which is why the probability of a worker’s productivity has to be recorded elsewhere. For this purpose, so-called market signals are used, which are understood as individual characteristics and achievements of employees, such as certificates acquired in the education system (Solga, 2008). Companies can use certificates from CVET to avoid bad investments so that ultimately the employees who have already been able to show corresponding certificates as a sign of their productivity participate in CVET. Another socioeconomic approach to explain the investment behavior of companies is provided by transaction cost theory (Williamson, 1988). The basic assumption of this theory is that, in negotiations between at least two contractual partners, costs such as information procurement costs or general negotiation costs are incurred. The level of information on the qualifications of employees on the company side can be regarded as high, whereas the quality of external workers can only be estimated with the help of additional expenditures (Lester, 2001). In line with this approach, companies with increasing shortages of skilled workers are expected to invest in employees who perform simple activities in order to secure internal demand and to increase labor loyalty to the company (Lester, 2001). Continuing vocational education and training of employees is also part of a corporate culture. Parameters of a corporate culture are common fundamental assumptions as the basis, shared values and norms of employees and leaders, and artifacts such as traditions and rituals (Schein, 1984). The corporate culture has measurable success on various factors, such as a higher identification with the company, better communication and conflict resolution ability of the employees, and fewer abstinence days due to illness (Sørensen, 2002). Taken together, these factors form an increased willingness to perform on the part of employees, which results in greater business success (Hartnell et al., 2011). An explanatory approach to individual efforts of further education activities gives the rational choice theory, which regards human behavior as the result of a choice between alternatives, whereby the respective actor chooses the best possible alternative for himself (Coleman and Fararo, 1992). The actor has resources and preferences for certain actions and is able choose between at least two alternatives in the decision-making situation. The core of any rational choice approach is the assumption that the actor selects the best possible alternative on the basis of situational circumstances, assessments, and information. Another theory that explains decisions is the expectation-value model (Rubenson, 1977; Gorges and Kandler, 2012). Especially for educational decisions, the Eccles model is frequently used (Eccles, 1983). In relation to individual efforts, the expectation-value theory comprises certain expectations of further education activities and its effects or results, which are weighed against the extent to which the requirements placed on CVET can be met (Gorges, 2016). According to Eccles (1983), factors that define value are individual learning experiences and affective memories (Gorges, 2016). Explicit determinants of value are, among other things, the benefits of achieving a professional goal or whether CVET brings pleasure. In contrast, there are negative influences such as fear of failure, effort, and monetary costs (Gorges, 2016).

Although there are different explanatory approaches, it can be subsumed that they rather offer reasons to participate or offer CVET for those persons who can already refer, for example, to a good educational background, named as the Mathew Effect (Merton, 1968). For those persons who do not have this basis, however, fewer reasons for participation in CET can be deduced. For this reason, it would appear helpful in the following to focus more on research findings on reasons and barriers that explicitly examine persons who are disadvantaged in CVET. In addition, research findings on the reasons for and barriers to offer CVET by companies as providers or supporters of CVET should also be presented.

## Empirical Findings on Reasons for and Barriers to Attending or Offering CVET by (Potential) Employees and Employers

### Reasons and Barriers From the Employees’ Point of View

The rate of employees who decide for or against participation in CVET differs according to sociodemographic characteristics, such as formal educational attainment (Bassanini et al., 2007; Desjardins, 2014). For example, CVET is more likely to be accepted by employees with a higher educational attainment. However, a different picture emerges in the area of individual CVET, where the number of low-formally qualified employees is higher. Low-formally qualified employees do not have a formal educational qualification at all or have only a lower secondary school leaving certification. Higher education qualifications include, for example, university degrees (Kyndt et al., 2011). Another sociodemographic factor that seems to influence the probability of CVET participation is age. With increasing age, the probability of participation increases but only up to a certain age, after which this effect decreases again. However, the sex of participants seems not to have an effect on participation in CVET (Bassanini et al., 2007).

Besides individual influences on participation in CVET, there are also sector-specific differences in participation rates. Industries with a rapid growth in technology and economy are especially dependent on CVET of their employees because current norms and procedures are subject to an accelerated aging process (Matukhin and Evseeva, 2014). In the hotel and catering sector, 24% of the total workforce in Germany participated in CVET in 2015. The participation rate was even at 28% in the retail sector (Wiseman and Parry, 2017). In the healthcare sector, an above-average number of CVET measures are promoted. In particular, medical and nursing service providers supported 24% of CVET activities for their employees. Another view is emerging in the retail sector; the portion of supporting companies has dropped to only 9% (Federal Institute for Vocational Education and Training, 2016).

When examining reasons to attend CVET, they can be summarized as the opportunity to improve professional performance and personal development by expanding and adapting skills, because CVET represents security of employability, offers career opportunities, and establishes social recognition and contacts (Krekel and Walden, 2007). Regarding barriers to attending CVET, Fertig and Huber (2010) refer to factors such as an unsuitable learning concept, a need for counseling, no suitable educational offer, and excessively high requirements. In addition, fears of not being able to adequately meet the expected workload or requirements represent a general barrier to attendance at CVET (Kilpi-Jakonen et al., 2015).

However, consideration of the reasons for and barriers to CVET across all employees, without taking into account the demographic characteristics just mentioned, including the sectors in which the individual is working, seems to be too shortsighted (for more details, see Siegfried et al., 2019). Thus, results show that, especially for older employees, the social aspect of CVET is a central reason for participation (Friebe and Schmidt-Hertha, 2013; Zwick, 2015). In general, older employees cite work-related reasons for participating in CVET less frequently than younger employees except when CVETs address organizational or technical changes (Behringer and Schönfeld, 2017).

For non-formally qualified employees, securing a qualification level and position in the company provides an opportunity for CVET. In addition, the obligation to participate in CVET often plays an important role (Kyndt et al., 2013; Behringer and Schönfeld, 2017).

In addition to career opportunities, regular participation in CVET is accompanied by an increase in income. However, the additional financial remuneration applies only to some groups. Studies show an influence of individual characteristics such as age or the formal qualification level of the employee. Thus, especially non-formally qualified employees benefit regarding income growth. Gender has no influence on salary (Wolter and Schiener, 2009).

Regarding barriers to attending CVET, studies outline that, for people re-entering work, a lack of necessity for further qualifications represents a major barrier to participation (Kilpi-Jakonen et al., 2015), whereas low-formally qualified employees cite limited time resources due to private or occupational obligations as an obstacle, especially if CVET is outside working hours (Kyndt et al., 2013). It is also pointed out that low-formally qualified employees are given fewer opportunities for participation by their employers, which in turn may result in the fact that they generally have less information about which CVET is suitable for them or which they could use to achieve their professional goals. Thus, when low-qualified employees are supported by their employees, they are much more likely to attend CVET (ebd.: Kuwan, 2002). Older employees cite a lack of support from the employer, a lack of prerequisites, and private or professional time restrictions as the reason for non-participation (Zwick, 2015). For employees with a migrant background, frequent barriers are a high financial burden and a low estimated benefit (Riesenfelder et al., 2011; Barz and Tippelt, 2018). Furthermore, non-formal requirements, such as certificates acquired abroad, represent a barrier for the disadvantaged groups (Kyndt et al., 2013).

### Reasons for and Barriers to Attending CVET by Jobseekers (Potential Employees)

For the group of jobseekers, and as potential employees, statistics for participation in CVET show that since 2012 the participation rate has stagnated by approximately 50% (Authoring Group Educational Reporting, 2016).

Most studies do not distinguish between employees and jobseekers when analyzing sociodemographic influences on further training behavior. However, this also means that formal qualifications, a migrant background, and age might be influencing factors for jobseekers as well. However, it should also be noted that people with lower formal qualifications are generally much more likely to be affected by unemployment than people with a higher qualification. Thus, the combination of unemployment and low qualifications increases the negative effect on participation in CVET (Van den Broeck et al., 2010; Käpplinger et al., 2013). This connection is partly explained by the negative learning experiences associated with low formal education, which in turn can lead to fears and resistance to CVET (Gellner et al., 2007).

If one examines the reasons of those who participate in CVET, both the improvement of professional opportunities and the prospect of a (new) job are given high priority (Chrisholm et al., 2004; Behringer and Schönfeld, 2017). In this context, studies show that participation in CVET is associated significantly with re-entry into employment and the associated return to gainful employment so that not only can an increasing financial independence be achieved (Rubenson and Desjardins, 2009). Moreover, the increasing skill requirements associated with a prospective new job encourage jobseekers to participate in CVET, which is principally aimed at expanding their knowledge and skills. In addition, technical changes and the resulting changes in work processes in many occupations result in the necessity for further qualifications and therefore the willingness to participate in CVET to get a certification or diploma (Chrisholm et al., 2004).

Beyond the requirements of the world of work and thus CVET measures, personal reasons for participation are also frequently cited (Chrisholm et al., 2004). For many jobseekers, personal satisfaction is a motivation to participate in CVET. Participation in CVET is associated with social participation and esteem. In this context, reasons to attend CVET refer to the establishment of social contacts and improvement in self-confidence (Chrisholm et al., 2004).

Reasons against participation in CVET among jobseekers and thus, in particular, among those groups of people who have no or low participation in CVET, primarily concern various implementation problems. These include the costs of CVET being too high or the non-existence of suitable CVET offers for jobseekers (Chrisholm et al., 2004). With regard to the first point regarding the costs of CVET, in addition to direct costs such as course fees and travel costs, indirect costs, in the form of learning efforts as well as opportunity costs resulting from lost alternative income, are also referred to as barriers to attending CVET (Gellner et al., 2007). Even if the barrier of opportunity costs is a rather short-term view, the long-term one, namely, that such training usually leads to an acknowledged vocational qualification or a partial qualification and thus opens up job opportunities, which in turn generate a higher income (Rubenson and Desjardins, 2009), jobseekers seem to be uncertain as to the extent to which these hoped-for financial benefits will result in higher wages and lower unemployment risk (Gellner et al., 2007). However, results show that the loss of leisure time and the direct costs seem to be a major factor (Gellner et al., 2007).

Another barrier to further education is the school-based learning environment, which is often associated with further education. The jobseekers, for example, state that they are no longer used to school-like learning and are afraid that they will not be able to meet the requirements (Rubenson and Desjardins, 2009). In this context, it is generally pointed out that fear of excessive demands has a negative influence on the probability of participation. Further barriers are mentioned in relation to family obligations, such as the care of children or relatives, but also one’s own health restrictions in the form of physical and mental illnesses (Chrisholm et al., 2004).

### Reasons for and Barriers to Offering CVET by Employers

From the point of view of an employer, one of the goals of CVET is to remain competitive (Desjardins, 2014; Zwick, 2015; Saar and Räis, 2017). As already mentioned in the introduction, CVET of existing workers offers great potential to close gaps in the filling of vacancies.

Thus, in 2012, the Federal Institute for Vocational Education and Training asked 2000 enterprises to what extent they thought they would encounter problems recruiting personnel in the next few years and, if so, whether they provide CVET or second-chance training to their older employees (Troltsch, 2012). Results show that overall only 45.9% of the enterprises are relatively certain that they will be confronted with problems regarding the recruitment of skilled employees. Of these companies, 8.5% offer second-chance training to young adults and CVET to older employees, whereas 10.9 and 10.1% provide only second-chance training or CVET for older employees, respectively. Interestingly, those companies generally accepting young adults without former vocational education and training, and therefore having the potential to offer second-chance training, are bigger companies with more than 200 employees, whereas the willingness to apply such CVET (second-chance training and/or CVET for older people) strategies decreases with the size of the company (Troltsch, 2012, p. 2–3). Other studies found this relation between the size of the company and the offer of CVET as well (Bassanini et al., 2007; Neubäumer and Kohaut, 2007; Walden, 2007; Brown and Sitzmann, 2011; Desjardins, 2014; CEDEFOP, 2015; Zwick, 2015; Saar and Räis, 2017). It is assumed that larger firms are more able to offer CVET than smaller firms because they have potentially more employees to send to a CVET, can offer to pay for CVET more easily, have larger and more diverse department structures to face changes more frequently, make learning processes more important, and enable the application of what has been learned.

Moreover, Troltsch (2012, p. 3; see also Zwick, 2005; Walden, 2007; Desjardins, 2014; Saar and Räis, 2017) outlines that, besides the size of a company, the sector of the company seems to play an important role regarding the extent to which CVET is offered. Results show that, for example, the retail sector, as well as the hotels and catering sector, offers significantly less CVET than the (high-)technology sector.

With respect to the reasons for and barriers to offering CVET (or not) mentioned by companies, they often cite the promotion of technical and also interdisciplinary competencies (e.g., reading or communication skills) of employees as a reason to offer CVET (Neubäumer and Kohaut, 2007; Walden, 2007; Allaart et al., 2009; Bellmann et al., 2014). Technical developments (e.g., new production processes or other technologically based changes) lead to changes at the organizational level with the result that new forms of organization and work have to be introduced, which in turn entail a shift of competencies and therefore additional CVET activities. Moreover, as was made clear at the beginning of this article, knowledge can be regarded as the fourth production factor due to increased knowledge intensity and the massive spread of information and communication technologies in many areas of work (e.g., Bassanini et al., 2007; Bellmann et al., 2014; CEDEFOP, 2015; Mönnig et al., 2019). Consequently, the (continued) existence of an enterprise also depends on the abilities and skills of its employees to correspond to current developments. Thus, in addition to safeguarding, the ability to innovate and develop knowledge, especially the adaptation of skills to new technologies, higher productivity, attractiveness as an employer, and an increase in the company’s social responsibility are also important aspects to be promoted by CVET in enterprises (Neubäumer and Kohaut, 2007; Saar and Räis, 2017).

On the other hand, high costs for CVET count as barriers to offering CVET (Walden, 2007; CEDEFOP, 2015). Walden (2007) found in his study that the costs of CVET differ regarding company size and industry sector. However, although larger companies seem to have higher training costs, because they are offering CVET more frequently, they are usually equipped with their own training department or full-time trainers, and they tend to provide training more frequently. Thus, the aspect of cost as a reason not to provide CVET might differ regarding company size and sector (Walden, 2007; see also Neubäumer and Kohaut, 2007; CEDEFOP, 2015).

Further barriers to offering CVET by the employer are that employees might leave the company after they have been trained and equipped with new skills, and therefore CVET is not worthwhile (Neubäumer and Kohaut, 2007; Saar and Räis, 2017). On the other hand, employers argue that the training expenditure exceeds the resultant benefit, in the form of an increase in qualifications, and that an investment is therefore not worthwhile for the company (Walden, 2007). However, the offer of CVET opportunities is also dependent on the employees themselves and to what extent they are willing to participate in CVET [see section “Reasons for and Barriers to Attending CVET by Jobseekers (Potential Employees)”]. Thus, the demand side has to be taken into account, as well when companies perceive only a limited need for CVET by their employees (Desjardins, 2014; Saar and Räis, 2017).

## Research Question

As has become clear from the previous theoretical and empirical considerations, the different perspectives with regard to employers and (potential) employees on CVET seem to address various as well as comparable reasons for and barriers to attendance at respective offers of CVET. Moreover, even if there are many gaps in the research, reasons for and barriers to attending CVET seem to differ regarding the individual characteristics of (potential) employees, as well as the institutional characteristics of the employer. Whereas the reasons and barriers mentioned by (potential) employees seem to depend on their different biographical and social circumstances, including previous educational biographies, age, longer interruptions in work, or migrant background, the reasons for and barriers to offering CVET by the employer seem to depend on firm size and sector. However, there are few findings available that compare both the view of the supply and demand side of CVET.

Based on rare previous results regarding individual and institutional characteristics and different approaches to explaining reasons for and barriers to attending and offering CVET, in the following empirical study, we look at three research questions:

(1) Why do employees, employers, and jobseekers attend CVET?
(2) To what extent do individual characteristics—older age, migrant background, lower qualifications—and institutional characteristics—company size and sector—matter with respect to the mentioned reasons for and barriers to attending CVET?
(3) How do (potential) employees’ and employers’ reasons for and barriers to attend or offer CVET differ?

## Materials and Methods

### Conduct of Semi-Structured Interviews and Sample Description

This complex interplay of individual characteristics and training behavior cannot be explained by simple causalities; rather, an in-depth analysis is required that employs a qualitative approach. Moreover, because there are no studies available that simultaneously examine reasons for and barriers to CVET from the perspective of employers, employees, and jobseekers, a qualitative approach using semi-structured interviews seems to be an appropriate way to answer the research questions. In contrast to the standardized questionnaires primarily used so far, these interviews allow an in-depth examination of the research object (Keegan, 2009; Saunders et al., 2009). The organization of semi-structured interviews enables comparable interview situations with variable adaptation and order of questions (Mayring, 2004). In order to ensure a uniform conduct of the interviews, interview training was carried out in advance. The interview guideline is based on between 7 and 10 questions (Table 1).

**TABLE 1:** Interview questions.

**(Potential) Employees**	**Employers**
(1) When was the last time you took part in in-company CVET measures? (2) Will you be taking part in CVET in the near future? (3) What significance does CVET have for you with regard to your professional and private life? (4) Why do you participate in CVET? (5) What are the reasons why you do not participate? (6) How do you imagine the ideal CVET in which you would definitely participate? (7) How do you become aware of CVET opportunities in your company?	(8) How important is in-company CVET in this company? (9) Do you offer CVET and, if so, which ones? (10) What causes your company to offer CVET? (11) What are the reasons why no CVET courses are offered? (12) Are CVET measures offered specifically for the groups of disadvantaged people we are looking at? Why are these offered or not offered? (13) What benefits does CVET bring to your company? (14) What experience have you had so far with in-company CVET in your company? (15) What challenges will in-company CVET have to face in your company in the future? (16) How do you communicate your CVET offer, especially to the groups of people we are looking at? (17) Which specific measures are taken to encourage employees to participate in CVET measures?

In total, 57 interviews were conducted with employers from different companies, 73 interviews with employees, and 42 interviews with jobseekers with differing demographic backgrounds. On average, employers working at small companies are 38.36 years old and 60% female, whereas employers working at bigger companies are 47 years old and only 45% female. A total of 47 female and 25 male employees were interviewed, with an average age of 42.97 years. The sample of jobseekers, on the other hand, consisted of 23 female and 19 male respondents with on average age of 40.14 years.

All in all, the interviewees were distributed very evenly among the retail sector (R) and healthcare and social services (H) sectors. For the hotel and catering sector (C) and (high) technology (T) sector, this was only possible for the employee and employer group; relatively few interviewees were found for the group of jobseekers in these sectors. Moreover, (potential) employees were differentiated according to the individual characteristics identified in the literature, namely, older people (people older than 50 years), migrant background (at least one parent or oneself was born abroad), no or low formal qualifications (persons without a lower secondary school leaving certificate and/or no vocational training qualification), and people re-entering work (people who had at least 1 year of career interruption) (for a more detailed overview of this grouping, see Seeber et al., 2017; Siegfried et al., 2019). For the group of jobseekers, it was not possible to interview a sample sufficiently differentiated according to the specified characteristics; thus, in the group of jobseekers, a distinction can only be made between jobseekers with a migrant background, jobseekers with a low or no formal qualifications, and older jobseekers. Furthermore, the data show that jobseekers were surveyed who did not show any of the identified disadvantageous characteristics. These are grouped together as “others.” In addition to those disadvantaged groups already identified in the literature, initial evaluation steps suggest for the sample of employees the formation of a further group in which certain characteristics cumulate. This applies here to employees with a migrant background and at the same time low or non-formal qualifications.

This is also quite consistent with the literature, which refers to the fact that persons with multiple disadvantages exhibit a different continuing training behavior (Bassanini et al., 2007) (Table 2).

**TABLE 2:** Sample characteristics.

			**Gender**			**Branch**			
		***N***	**Female**	**Male**	**Age (*SD*) (y)**	**Retail**	**Healthcare**	**Catering**	**Technology**
Employee	Migration background	14	5	9	31.29 (8.20)	3	–	8	3
	Migration background and low qualification	21	10	11	35.95 (11.05)	7	5	5	4
	Older employees	26	20	6	56.27 (4.22)	6	11	5	4
	People re-entering work	12	12		39.83 (6.52)	5	4	2	1
	Sum	72	47	25	42.97 (12.94)	21	20	19	12
Employer	Companies with <500 employees	15	9	6	38.36 (10.26)	4	7	4	–
	Companies with >500 employees	35	15	18	47 (10.55)	9	7	13	6
	Sum	57	37	28	47.04 (10.22)	16	15	20	6
Jobseeker	Migration background	11	6	5	35.36 (0.45)	3	4	2	
	Low (formal) Qualification	7	4	3	30.14 (0.43)	3	1	1	1
	Older people	11	7	4	55.55 (0.36)	2	3	–	1
	Others	13	6	7	36.54 (8.69)	3	3	2	3
	Sum	42	23	19	40.14 (11.74)	11	11	5	5

### Categorization and Analysis of the Interviews

The interviews were transcribed, and a qualitative content analysis using a deductive and inductive category system, with definitions of the category and anchor examples, was used for their systematic evaluation (Mayring, 2004). Already known theories and empirical results on the reasons for and barriers to CVET participation by employees and jobseekers and the reasons for and barriers to offer CVET on the part of employers formed the basis of the theory-guided deductively developed categories. As a second step, new categories were inductively derived from the interviews (for a more detailed description, see Siegfried et al., 2018, 2019). The resulting coding guidelines consist of 15 upper categories, divided into reasons and barriers (see Table 3 for an excerpt).

**TABLE 3:** Overview of categories.

**Reasons**	**Barriers**
**Work-related**	
Obligation to participate	Organizational by employer or CVET-institute
Receipt of a certificate	Inappropriate CVET offer
Professional networking/professional exchange	No sense of need/perceive limited demand
Ensuring the current professional situation (e.g., due to more complex tasks)	Economic efficiency/added value of CVET unclear
Change or extension of activities at work (e.g., induction training)	Costs of CVET
Change of products/services/work equipment	Occupational concurrent obligations
Structural reorganization in the company	
Desired professional change/increase job opportunities/strategic personnel development	
Refreshing/protection of existing job-related knowledge/enhancement of employee skills	
Responsibility toward the company (e.g., securing competitiveness)	
Additional financial compensation	
Corporate and leadership culture	
Assumption of costs by employer/CVET-institute	
**Not directly work-related**	
Interest in the topic	Physical/psychological restrictions
Further development/maintenance of interdisciplinary knowledge	Private concurrent obligations
	Convenience of (potential) employee

To ensure a high coding quality, 20% of the interviews were initially coded by a team of two coders. After achieving a satisfactory interrater reliability (Cohen κ from 0.61 to 0.78), subsequent coding was done individually.

## Results

### Reasons to Attend and Offer CVET

The analyses of reasons for and barriers to attendance at CVET, with respect to their relevance between the different disadvantaged groups and in particular between the (potential) employee and employer perspectives, were carried out descriptively. In a next step, an exact test according to Fisher was used^1^ to determine whether the sector and affiliation to a specific disadvantaged group had a significant effect on the distribution of the naming of reasons and barriers to attend CVET (Table 4). Because the employee group of the low and/or non-formally qualified employees consists of only four persons, after the formation of the disadvantaged group with the multiple disadvantages of migrant background and low and/or non-formal qualifications and thus for whom no reliable statements are possible, only four of the disadvantaged groups were used for the following analyses: (1) employees with a migrant background (MB), (2) employees with a migrant background and low/non-formal qualifications (MB + LQ), (3) employees re-entering work (RW), and (4) older employees (O).

**TABLE 4:** Reasons to attend and offer CVET.

**Actor**	**Employees**									**Jobseekers**					**Employer**							
**Groups**	**M**	**Target groups**				**Branch**				**M**	**Target group**				**M**	**Branch**				**Size**		
**Reasons**		**MB**	**O**	**RW**	**MB+LQ**	**R**	**H**	**C**	**T**		**Others**	**MB**	**LQ**	**O**		**R**	**H**	**C**	**T**	**KMU**	**GU**	**Fisher’s Exact Test**
*Work-related*																						
Obligation to participate	**29%**	23%	38%	17%	29%	29%	40%	32%	8%	**19%**	15%	18%	14%	27%	**53%**	38%	67%	50%	67%	53%	57%	*p* = 0.001
**Fisher’s Exact Test**		n.s.				n.s.						n.s.				n.s.				n.s.		
Receipt of a certificate	**19%**	31%	4%	8%	29%	14%	15%	21%	33%	**79%**	85%	64%	86%	82%								*p* < 0.001
**Fisher’s Exact Test**		*p* = 0.004				n.s.						n.s.				n.s.				n.s.		
Professional networking / professional exchange	**24%**	8%	23%	42%	29%	48%	15%	5%	25%	**5%**	8%	0%	14%	0%								*p* = 0.009
**Fisher’s Exact Test**		n.s.				*p* = 0.012						n.s.										
Ensuring the current professional situation	**24%**	38%	12%	42%	12%	19%	15%	21%	50%						**19%**	25%	0%	25%	33%	13%	20%	
**Fisher’s Exact Test**		*p* = 0.05				n.s.										n.s.				n.s.		
Change or extension of activities at work	**32%**	46%	38%	33%	12%	19%	30%	37%	50%						**42%**	69%	13%	35%	67%	20%	54%	
**Fisher’s Exact Test**		n.s.				n.s.										*p* = 0.007				*p* = 0.033		
Change of products/services/work equipment	**76%**	54%	73%	92%	88%	90%	65%	68%	83%						**58%**	69%	53%	40%	100%	53%	63%	*p* = 0.036
**Fisher’s Exact Test**		n.s.				n.s.										*p* = 0.044				n.s.		
Structural reorganization in the company	**0**	0	0	0	0	0	0	0	0						**11%**	13%	7%	5%	33%	7%	14%	*p* = 0.006
**Fisher’s Exact Test**		n.s.				n.s.										n.s.				n.s.		
Desired professional change/increase job opportunities/strategic personnel development	**33%**	54%	19%	33%	35%	38%	15%	47%	33%	**83%**	92%	73%	71%	91%	**75%**	81%	80%	65%	83%	67%	83%	*p* < 0.001
**Fisher’s Exact Test**		n.s.				n.s.						n.s.				n.s.				n.s.		
Refreshing/protection of existing job-related knowledge/enhancement of employee skills	**28%**	15%	12%	67%	35%	43%	20%	32%	8%						**81%**	94%	100%	55%	83%	93%	77%	*p* < 0.001
**Fisher’s Exact Test**		*p* = 0.007				n.s.										*p* = 0.002				n.s.		
Responsibility towards the company	**13%**	15%	8%	8%	24%	14%	5%	16%	17%						**53%**	94%	27%	30%	83%	40%	60%	*p* < 0.001
**Fisher’s Exact Test**		n.s.				n.s.										*p* < 0.001				n.s.		
Additional financial compensation	**13%**	23%	4%	8%	24%	10%	0%	0%	58%	**10%**	15%	9%	0%	9%								
**Fisher’s Exact Test**		n.s.				*p* < 0.001						n.s.										
Corporate and leadership culture	**31%**	23%	35%	25%	41%	38%	25%	21%	42%						**58%**	81%	60%	40%	50%	60%	54%	*p* = 0.002
**Fisher’s Exact Test**		n.s.				n.s.										n.s.				n.s.		
Assumption of costs by employer/CVET-Institute	**11%**	8%	15%	17%	6%	0%	20%	11%	17%	**68%**	77%	60%	86%	55%								*p* < 0.001
**Fisher’s Exact Test**		n.s.				n.s.						n.s.										
*Not directly work-related*																						
Interest in the topic	**53%**	46%	62%	58%	47%	43%	60%	42%	75%	**38%**	31%	27%	43%	55%								
**Fisher’s Exact Test**		n.s.				n.s.																
Further development/maintenance of interdisciplinary knowledge	**82%**	85%	88%	100%	65%	90%	95%	68%	67%	**55%**	77%	55%	43%	36%	**47%**	75%	40%	25%	67%	53%	46%	*p* < 0.001
**Fisher’s Exact Test**		*p* = 0.038				n.s.						n.s.				*p* = 0.016				n.s.		

*R = Retail; H = Healthcare; C = Catering, T = Technology; MB = Migrant background; RW = People reentering work; O = Older worker; MB+LQ = Migrant background and low qualification. M = Mean value; bold values represent the mean values for a considered group per category.*

The refreshing of existing job-related knowledge due to a desired increase in the abilities of the employee seems to be the most important reason for employers to offer CVET. However, while almost all employers, and differentiating between the sectors, especially employers in the high-technology and retail and hotel sectors (R: 94%; H: 100%; T: 83% in comparison to C: 55%), cite this as a reason to offer CVET, this is only the case for an average of 30% of employees. However, it must be pointed out that there are significant differences between the different disadvantaged groups of employees as well (*p* = 0.004). Thus, the lower importance of this reason came from employees with a migrant background (15%), older employees (12%), and employees with a migrant background and low qualifications (35%), whereas employees re-entering work mention this reason significantly more often (67%).

Obtaining a certification plays another very important role in employers offering CVET (79%) and in jobseekers attending CVET (79%). In comparison, employees mention this reason significantly less, although significant differences between the disadvantaged groups of employees must be taken into account (*p* = 0.004). Thus, mainly employees with a migrant background, as well as employees with a migrant background and low qualifications, mention this reason (MB: 31%; MB + LQ: 29% in comparison to the other disadvantaged groups: RW: 8%; O: 4%).

The third most important reason to offer CVET according to employers (75%) and jobseekers (83%) is filling vacancies strategically; for jobseekers, this increases the possibility of getting a job. In contrast, on average, only 33% of employees, independent of belonging to a disadvantaged group or sector, give this reason.

Moreover, responsibility toward the company (e.g., securing competitiveness), the corporate and management culture, strategic professional development, and the obligation to participate in CVET appear to be further important reasons for employers to offer CVET. Even though there are significant differences between the different sectors with regard to responsibility toward the company (*p* < 0.001; R: 94%; T: 83% in comparison to H: 27%; C: 30%), all three reasons are mentioned significantly more often by employers in comparison to employees and jobseekers (responsibility: *p* < 0.001; culture: *p* = 0.002; strategic professional development: *p* < 0.001).

Taking the most important reasons mentioned by the jobseekers into account in addition to the already mentioned certification (79%) and desired increase in job opportunities (83%), the assumption of costs by an institute is also an important reason (68%). It is interesting to note that this is also an important reason for jobseekers, but not for employees (on average, 11% of employees mention this reason).

In looking at the most important reasons to attend CVET according to the employees, the further development or maintenance of interdisciplinary knowledge is mentioned by on average 82% of employees, although there are significant differences between the disadvantaged groups (*p* = 0.038; MB: 85%; O: 88%; RW: 100%; MB + LQ: 65%). Employers in the healthcare and catering sectors (H: 40%; C: 25% in comparison to R: 75%; T: 67%) and jobseekers (55%) mention this reason significantly less (*p* < 0.001). Another very important reason for employees to attend CVET can be seen in the change in products, services, and work equipment (mentioned on average by 76% of employees across all sectors and disadvantaged groups). In comparison, on average, 58% of employers mention this reason. However, there are significant differences between the sectors in which the employers are working (*p* = 0.044; R: 69%; H: 53%; C: 40%; T: 100%). Thus, significant differences between employers and employees are only significant for the healthcare and catering sectors. The third most important reason to attend CVET reflects the interest in the topic, which is mentioned with no significant difference by employees (53%) and jobseekers (38%).

Other reasons to attend CVET seem to be less important because they are mentioned on average by approximately 30% of (potential) employees or employers. However, results show significant differences between those two actors. This is the case for the possibility of vocational networking or professional exchange. This reason plays a more important role for employees (24%) than for jobseekers (5%; *p* = 0.009). However, these results have to be distinguished between the different sectors in which employees are working (*p* = 0.012) because employees from the technology sector in particular mention this reason for attending CVET (T: 25% in comparison to R: 14%; H: 15%; C: 5%). The intention of additional financial remuneration due to participation in CVET is often cited only by persons from the technical sector (58%) as a reason to attend CVET, whereas employees in other sectors (R: 10%, H: 0%; C: 0%) and jobseekers (10%) rarely cite this reason. No significant difference between employees and employers can be found for the reason “ensuring the current professional situation.” However, significant differences between the disadvantaged groups have to be taken into account (*p* = 0.05; MB: 38%; RW: 42%; MB + LQ: 12%; O: 12%).

Structural reorganization in a company is mentioned only by employers and not by employees as a reason to attend CVET. However, only a small proportion of employers mentioned this reason (11%).

Only two reasons without any significant difference between employers and employees can be identified, namely, the change or extension of activities at work and ensuring the current professional situation. On average, 31.9% of employees consider the change or extension of activities to be a factor to attend CVET, whereas the relevance of this event differs significantly between employers groups in relation to the sector (*p* = 0.007; R: 69%; H: 13%; C: 35%; T: 67%) and company size (*p* = 0.033; companies with <500 employees: 20%; companies with >500 employees: 54%). This means that employers in the retail and technology sectors and large companies, in particular, see this as an important reason to participate in CVET.

### Barriers

Regarding the identified barriers (Table 5), the barrier to offering CVET mentioned by most of employers refers to competitive occupational obligations (54%). Moreover, on average, 30% of all employees mention this barrier, but for the jobseekers, this barrier is hardly relevant (12%; significant differences between the actors: *p* < 0.001). The costs of CVET are another important barrier to offering CVET for employers (46%) and jobseekers (38%) but not so much for employees (10%). However, significant differences regarding the disadvantaged groups of jobseekers have to be taken into account (*p* = 0.022). Results demonstrate that this barrier is mentioned by only 9% of jobseekers with a migrant background, 36% of older jobseekers, 29% with a low qualification background, and 69% with no disadvantageous characteristics. A third important barrier that leads to the situation of no CVET being offered by employers refers to the limited demand for CVET (37%). Employees, on the other hand, are interested in CVET (see section “Barriers”), but would like to see more suitable CVET offers because most of them mention the problem of no suitable CVET as a barrier (50%) in contrast to jobseekers (32%) and employers (21%).

**TABLE 5:** Barriers to attending and offering CVET.

**Actor**	**Employees**									**Jobseekers**					**Employer**							
**Groups**	**M**	**Target groups**				**Branch**				**M**	**Target group**				**M**	**Branch**				**Size**		
**Barriers**		**MB**	**O**	**RW**	**MB+LQ**	**R**	**H**	**C**	**T**		**Others**	**MB**	**LQ**	**O**		**R**	**H**	**C**	**T**	**KMU**	**GU**	**Fisher’s Exact Test**
*Work-related*																						
Organizational by employer or CVET-institute	**10%**	0%	19%	0%	12%	14%	5%	0%	25%	**2%**	8%	0%	0%	0%	**25%**	31%	27%	25%	0%	53%	11%	*p* = 0.003
**Fisher’s Exact Test**		n.s.				n.s.						n.s.				n.s.				*p* = 0.003		
Inappropriate CVET offer	**50%**	46%	58%	58%	29%	48%	60%	37%	58%	**33%**	15%	36%	29%	55%	**21%**	13%	33%	25%	0%	27%	17%	*p* = 0.003
**Fisher’s Exact Test**		n.s.				n.s.						n.s.										
No sense of need/perceive limited demand	**11%**	23%	4%	0%	24%	14%	0%	16%	17%						**37%**	44%	47%	20%	50%	33%	40%	*p* < 0.001
**Fisher’s Exact Test**		n.s.				n.s.						n.s.				n.s.				n.s.		
Economic efficiency/added value of CVET unclear										**5%**	15%	0%	0%	0%	**16%**	19%	13%	10%	33%	13%	14%	
**Fisher’s Exact Test**												n.s.				n.s.				n.s.		
Costs of CVET	**10%**	8%	4%	0%	24%	10%	0%	11%	17%	**38%**	69%	9%	29%	36%	**46%**	56%	47%	30%	67%	40%	46%	*p* < 0.001
**Fisher’s Exact Test**		n.s.				n.s.						*p* = 0.022				n.s.				n.s.		
Occupational concurrent obligations	**33%**	23%	31%	25%	47%	24%	40%	32%	42%	**12%**	23%	0%	29%	0%	**54%**	63%	47%	50%	67%	53%	57%	*p* < 0.001
**Fisher’s Exact Test**		n.s.				n.s.						n.s.				n.s.				n.s.		
*Not directly work-related*																						
Physical / psychological restrictions	**21%**	0%	19%	50%	18%	33%	20%	11%	17%	**24%**	23%	27%	0%	36%								
**Fisher’s Exact Test**		*p* = 0.036				n.s.						n.s.										
Private concurrent obligations	**32%**	38%	19%	67%	29%	43%	25%	21%	42%	**14%**	8%	27%	29%	0%								*p* = 0.045
**Fisher’s Exact Test**		*p* = 0.036				n.s.						n.s.										
Convenience of (potential) employee	**15%**	8%	12%	17%	24%	0%	20%	11%	42%	**0%**	0%	0%	0%	0%								*p* < 0.001
**Fisher’s Exact Test**		n.s.				*p* = 0.008					n.s.											

*R = Retail; H = Healthcare; C = Catering, T = Technology; MB = Migrant background; RW = People reentering work; O = Older worker; MB+LQ = Migrant background and low qualification. M = Mean value; bold values represent the mean values for a considered group per category.*

Private concurrent obligations also represent a barrier to attending CVET for employees (32%) significantly more often than for jobseekers (*p* = 0.045; 12%), although this result has to be distinguished between the disadvantaged groups (*p* = 0.036; RW: 67% in comparison to MB: 38%; MB + LQ: 29%; O: 19%).

The individual convenience for the employee by whom an additional possible load in the form of CVET is avoided is addressed significantly different regarding the sectors in which employees work (*p* = 0.008). While 42% of those surveyed in the high-technology sector name this barrier, the proportion in the other sectors is between 0 and 20%. The jobseekers do not name this barrier at all. The barrier of the uncertainty of the economic efficiency and added value from CVET is also rarely mentioned by both the jobseekers (2%) and the employers (16%).

## Discussion

The article used the current structural change in the labor market, technological, structural, and demographic developments and the resulting need for preventive investment in education as an opportunity to conduct a comprehensive interview study to analyze the various reasons for and barriers to employees attending CVET and employers offering CVET. Continuing vocational education and training not only offer the prospect of increasing individual employment opportunities, but it also affords the opportunity to overcome the shortage of skilled labor by activating previously unused labor potential. The comparison of potential similarities and also discrepancies between the (potential) employees’ and the employers’ perspective on CVET, which is the aim of this article, makes a valuable contribution in that it (1) on the one hand, patterns of participation can be identified, particularly by those groups of employees who have received less consideration in CVET (see section “Differential Explanatory Approaches to Offering and Participating in CVET”); (2) on the other hand, the inclusion of the employer’s role and the reasons for and barriers to CVET is a first step to bring the two central actors for CVET together. Even though this is an exploratory approach, which has not been found in the literature in this form up to now, the insights gained can give first hints on which incentive structures can be created [for (potential) employees and employers] but also which are the barriers shared by (potential) employees and employees that need to be dismantled, for example, through state intervention.

First, results indicate that, in addition to sectoral differences (and differences between the disadvantaged groups) for employees and employers, a comparison of the reasons for offering CVET with the reasons why employees participate in CVET shows sometimes considerable differences as well. It is, moreover, interesting that the sector-specific reasons identified for employees are different from the target group-specific reasons. If (potential) employees are also included in this analysis, it becomes clear that greater differences between employees and employers or jobseekers emerge, while hardly any differences between jobseekers and employers can be identified. These discrepancies between employees and employers are gaining importance against the background of incentives to participate in CVET that are incorrect or not established. With regard to the barriers to attend or to offer CVET, it is particularly important to highlight, on the one hand, those barriers that are highly relevant for both employees and employers, because they are important but especially difficult to overcome and, on the other hand, those differing between these two actors, because they might be particularly easy to remove.

By focusing in a first step on reasons and therefore potential incentives to attend or offer CVET, it becomes clear that employees participate in CVET mainly if it enables them to react to new requirements in their field of work (e.g., change in products, services, and work equipment: *M* = 76% in comparison to employers: *M* = 58%). In comparison, employers primarily address the refreshment or promotion of the professional competence of their employees, such as refreshing/protection of employee skills, strategic personnel development, change of products/services/work equipment, and responsibility toward the company in the CVET measures when they mention reasons to offer CVET. However, sector-specific differences have to be taken into account, because the retail and technology sectors, in particular, and occasionally also the healthcare sector, mention these reasons for offering CVET particularly frequently. While these reasons for offering CVET by employers seem to address the maintenance of internal human capital of the company, the attendance of CVET by employees can be referred to the rational choice approach. The employees are facing a situation of changing working conditions and have the possibility to meet these requirements by attending a CVET or no longer be able to carry out the work process satisfactorily. The low costs of workplace CVET, as these are mainly paid for by the employers (CEDEFOP, 2015), are thus offset by a relatively large benefit in the form of further abilities to act in the job. This can also explain why simply refreshing or protecting of existing job-related knowledge or a desired professional change/strategic personnel development—which are as already mentioned as the most important reasons for employers to offer CVET—is hardly a reason for employees to participate in CVET. Here, the costs of CVET in terms of time spent are offset by a relatively small benefit or an unsecure benefit. Referring to the less mentioned reason of refreshing or protecting of existing job-related knowledge, most employees have already been working in this area of activity for some time, so minor changes can be adapted quickly. However, this explanation must be distinguished with regard to the disadvantaged group. Thus, employees returning to work after a longer break cite this reason for CVET very frequently (*M* = 67%); refreshing their knowledge helps them to be able to resume work, so the benefits seem to be assessed higher than the costs. In the case of a desired professional change/strategic personnel development, the uncertainty about the potential benefits and which CVETs make it easier to change jobs does not appear to directly relativize the costs of participation in CVET. However, here again, group differences among employees and the viewpoint of jobseekers must be taken into account, because employees with a migrant background (*M* = 54%) and jobseekers (*M* = 83%) cite this reason for participating in CVET relatively frequently. In connection with the reason to obtain a certificate, which is also often named by these (potential) employees—in contrast to the other groups of employees—there is the presumption that CVET is used as a signal of their market value. This can be explained by the existing recognition problems with previous foreign educational pathways or due to lower formal educational qualifications that are often linked to a migrant background or unemployment.

Another frequently mentioned reason to attend CVET refers to further develop/maintain interdisciplinary knowledge, whereas a change of job or task is a CVET reason for only a few employees. This can be explained by the expectation-value theory, because often it is not clear which CVET exactly has to be attended for a promotion or job change, so the value of a job change in connection with the uncertainty regarding whether it can be guaranteed with the CVET lowers the expectation value. Interestingly, interdisciplinary competencies also represent an important reason to offer CVET according to most employers (more precisely employers in the retail and technology sector); this cannot be sufficiently explained by the human capital theory because there is a possibility that trained employees can use their potential in another company. An explanation could be the avoidance of transition costs or the increasing difficulty to find skilled workers in the labor market. Thus, employers, regardless of their specificity, generally show a higher willingness to train further.

The reasons already described for employees’ participation in CVET differ only in terms of disadvantaged groups. However, for the less frequently mentioned reason of professional exchange through CVET or additional financial compensation, there are differences in relation to the sector. While employees in the technology sector mention this reason much more frequently (*M* = 48%) in comparison to employees in the other sectors, there are no significant differences in relation to the target group.

In summary, it becomes clear that some reasons to offer CVET, such as the change in products, services, and work equipment or further develop/maintain of interdisciplinary knowledge, which are already frequently cited by employers as a reason for offering CVET, could be even more focused by employers, because those reasons address important reasons to participate in CVET by employees too. Thus, the creation of a work culture that allows or explicitly strives for innovation in production and service and work equipment can offer a good incentive structure for employees to participate in CVET. In turn, the company would also benefit from this, as it would at least maintain its own interests, that is, its ability to innovate, if not expand it. On the other hand, additional financial incentive structures or certified qualification measures can offer the possibility to target specific groups of employees or employees in specific sectors.

Taking in a second step mentioned barriers into account, results show that costs for employers and jobseekers often constitute a barrier to offering CVET perhaps due to fears of failure and uncertainty about possible success (to keep employees in the company or for jobseekers to find a job). Moreover, jobseekers depend on unemployment pensions, which represent only a low level of state support. Thus, financial resources are limited; therefore, individual financing of CVET is hardly possible, and participation in CVET is bound to the financial support of the supervising institute. For employees, however, costs are hardly a reason because the costs of workplace CVETs in particular are often paid by companies (CEDEFOP, 2015). Therefore, the solution to overcome the barrier of costs according to employers might be to pass the costs on to employees. However, because previous studies refer to cost as an important barrier for employees (Riesenfelder et al., 2011; Barz and Tippelt, 2018), the benefit of this solution in increasing the attendance of disadvantaged employees is questionable. Thus, if employers want their employees to participate more in CVET measures, and if the inclusion of jobseekers becomes more relevant due to the lack of skilled workers, it seems necessary that (1) government funds are made available to support the integration of jobseekers into the labor market, and (2) the costs of CVET must be reduced. Blended learning or e-learning approaches offer one possibility here, because the costs of trainers can be reduced, and as the training can be open to a larger number of participants, here again costs can be reduced. On the other hand, temporal and spatial flexibility facilitates the linkage of work and learning processing, which may in turn be used to overcome the barrier of occupational obligation, defined by both employers and employees as an important barrier. The learning object through blended or e-learning is segmented into learning units that build on one another and are offered sequentially so that it is possible to interrupt processing at any time and resume it at a later point in time.

## Limitations and Future Research

First, it should be noted that, even if CVET is seen in the literature as an important way to meet the aforementioned challenges, focusing on the reasons for and barriers to participate or offer CVET does not initially take account of research work and its findings on the impact of CVET on learning outcomes and its adoption in the job (see, e.g., Hinrichs, 2014; Sandmeier et al., 2018; Harteis et al., 2020). Thus, when it comes to the actual impact of CVET on the mentioned challenges, its consideration in further studies is therefore essential. At the same time, however, a CVET offer tailored for (potential) employees and the identification of relevant participation patterns to enable this adequate CVET offer by employers are required first in order to take a closer look at the potential of learning transfer in a second step.

Moreover, it must be pointed out that in the study only those persons who volunteered to take part were interviewed. Therefore, a corresponding positive selection, that is, that mainly persons participating in CVET were interviewed, cannot be ruled out. It can therefore be assumed that, with regard to the barriers mentioned, few findings of persons who (in principle) do not attend CVET are included. However, the test persons were asked about possible barriers that may have caused them to reduce the extent of their CVET activities. Thus, the results of the study focus more on barriers that reduce the extent of participation but may not entirely prohibit participation. However, it would be interesting to ask the opinions of those who have indicated no interest in participating.

Furthermore, for the quantification of reasons and barriers, only the information as to whether a person names the reason or barrier was used, and this was viewed in relation to a group of persons. This made it possible to analyze to what extent many or only individual persons in a group named the same reason or barrier and therefore to what extent this reason or barrier was important to the whole group. However, information about the significance of the reasons or barriers for individual persons has been lost.

Results, moreover, are based on a qualitative approach. Even if the number of people surveyed permits initial analyses with regard to the identification of significant differences in the mentioned reasons and barriers regarding the groups investigated, a much larger sample is required in order to be able to make reliable statements (see the contribution of Lischewski et al., in revision). In this context, quantitative studies based on the available results should be encouraged, which explicitly address the differences found in this study in order to evaluate the general validity. The focus should be on the reasons and barriers that are contrary to previous studies (e.g., in this study, costs of CVET are not barriers for employees) and/or focus particularly on those reasons and barriers that differ between employer and employee (e.g., limited demand, unsuitable CVET supply) or that make the offer of and attendance at CVET challenging (e.g., competing occupational obligations). Information gained in this way can be used to address specific CVET offerings. This procedure seems particularly important in light of the current challenges.

## Data Availability Statement

The datasets generated for this study are available on request to the corresponding author.

## Ethics Statement

Ethical review and approval was not required for the study on human participants in accordance with the local legislation and institutional requirements. The patients/participants provided their written informed consent to participate in this study.

## Author Contributions

CS and JB contributed to the conception and the design of the work. CS did the analysis and interpretation of the data. CS and JB wrote the manuscript. CS and JB agreed all aspects of the work and approved the final version of the article to be published.

## Conflict of Interest

The authors declare that the research was conducted in the absence of any commercial or financial relationships that could be construed as a potential conflict of interest.

## Footnotes

1 Because the sample for some of the groups’ forms (e.g., employer in the technology sector or low qualified jobseekers) is rather small, and the expected frequencies in some cells are less than five, the χ^2^ test approximation becomes inaccurate (Field, 2013, p. 723).
